# A dynamic method for the investigation of induced state metabolic capacities as a function of temperature

**DOI:** 10.1186/1475-2859-12-94

**Published:** 2013-10-15

**Authors:** Patrick Sagmeister, Timo Langemann, Patrick Wechselberger, Andrea Meitz, Christoph Herwig

**Affiliations:** 1 Institute of Biochemical Engineering, Vienna University of Technology, Vienna, Austria; 2 Research Center of Pharmaceutical Engineering (RCPE) GmbH, Graz, Austria; 3 BIRD-C GmbH, Kritzendorf, Austria; 4 CD Laboratory for Mechanistic and Physiological Methods for Improved Bioprocesses, Vienna, Austria

**Keywords:** Bioprocess technology, Fermentation, Dynamic experiments, Process control, Soft-sensors

## Abstract

**Background:**

Science-based recombinant bioprocess designs as well as the design of statistical experimental plans for process optimization (Design of Experiments, DoE) demand information on physiological bioprocess boundaries, such as the onset of acetate production, adaptation times, mixed feed metabolic capabilities or induced state maximum metabolic rates as at the desired cultivation temperature. Dynamic methods provide experimental alternatives to determine this information in a fast and efficient way. Information on maximum metabolic capabilities as a function of temperature is needed in case a reduced cultivation temperature is desirable (e.g. to avoid inclusion body formation) and an appropriate feeding profile is to be designed.

**Results:**

Here, we present a novel dynamic method for the determination of the specific growth rate as a function of temperature for induced recombinant bacterial bioprocesses. The method is based on the control of the residual substrate concentration at non-limiting conditions with dynamic changes in cultivation temperature. The presented method was automated in respect to information extraction and closed loop control by means of in-line Fourier Transformation Infrared Spectroscopy (FTIR) residual substrate measurements and on-line first principle rate-based soft-sensors. Maximum induced state metabolic capabilities as a function of temperature were successfully extracted for a recombinant *E. coli* C41 fed-batch bioprocess without the need for sampling in a time frame of 20 hours.

**Conclusions:**

The presented method was concluded to allow the fast and automated extraction of maximum metabolic capabilities (specific growth rate) as a function of temperature. This complements the dynamic toolset necessary for science-based recombinant bacterial bioprocess design and DoE design.

## Background

Today, biopharmaceuticals are the principal driver of innovation in the pharmaceutical industry [1,2]. Fuelled by regulatory initiatives [3] as well as the emerging focus on biosimilars (follow-on biologics), novel strategies for the science and risk based development of efficient pharmaceutical bioprocesses are needed.

Bioprocess development aims at the identification and quantification of the interactions of process parameters with productivity and product quality related attributes, typically with the goal of process optimization. Enhanced bioprocess development approaches following quality by design (QbD) principles additionally aim at the demonstration of process understanding. Following a science and risk based approach, the impact of parameters critical in respect to final product quality (critical process parameters, CPPs) on attributes in relation to final product quality (critical quality attributes, CQAs) is systematically analyzed, providing a high degree of insight in the process under investigation. The scientifically developed process understanding can then be communicated to the regulatory authorities with the benefit of increased manufacturing flexibility [3].

The basic toolset for QbD driven process development as discussed by several recent contributions [4-10] and covered in recent text books [4,11] includes following elements:

1) Risk assessment approaches for the identification of possible CPPs and CQAs

2) Process analytical technology (PAT) methodologies that allow to “design, analyze and control manufacturing through timely measurements”

3) Design of experiments (DoE), a statistical approach aiming at the systematic and efficient investigation of the input factors’ (for example CPPs) impact on defined responses (for example CQAs).

### Specific growth rate μ and cultivation temperature: Key parameters governing bioprocessing efficiencies

Product formation rates in fed-batch processes are primary determined by the specific growth rate (μ) [12-17] or respectively the specific substrate uptake rate qs [18-20], which are therefore of primary interest for the design and investigation of recombinant bioprocesses. Both variables are typically controlled via open loop feeding trajectories (exponential feeding), or closed-loop feedback controllers [21,22] and are directly interlinked via the biomass yield coefficient.

Next to the specific growth rate μ, cultivation temperature has been in primary focus of bioprocess development. Rationales for including temperature as factor are to increase the yield of active recombinant protein through the increase of solubility [23-25], more efficient folding through slower protein expression [26,27], less self-association of recombinant products [28], decrease of protease activity [29] as well as the possible impact of temperature on plasmid stability [30].

Due to the importance of cultivation temperature as well as specific growth rate μ in respect to productivity and product quality, these factors are of primary interest for basic bioprocess design as well as bioprocess optimization. In order to do so, information on physiological boundaries is necessary: The maximum specific growth rate at a given temperature is not to be exceeded; otherwise experimental plans fail (desired growth rate is not achieved) and substrate accumulates. Hence, information on the maximum growth rate μ as a function of temperature is needed for basic process design and DoE design purposes.

Dynamic fed-batch experimentation refers to the dynamic deflection of (physiological) process states for the fast extraction of physiological information such as i) information on overflow metabolism [22,31], ii) adaptation to novel substrates [32] and iii) maximum metabolic capabilities of the system under investigation and even iv) productivities in dependence to physiological states. Dynamic methods for bioprocess design were reviewed recently by Spadiut et al. [33]. However, to the authors’ knowledge there has been no dynamic study so far dealing with the determination of the temperature dependency of the specific growth rate.

Temperature adaptation of *E. coli* is reported to be very fast [34]. Therefore, quasi steady states in fed-batch experiments can be assumed and the application of dynamic ramp methods is appropriate. Hence, the application of dynamic experiments to determine the relationship of μ and temperature should be feasible.

### Real-time bioprocess monitoring

Information on chemical, biological and physiological process variability is highly desired for bioprocess analysis (identify and allocate sources of variation), measurement of CPPs and CQAs, real-time event detection (e.g. depletion of a carbon source) and bioprocess control. Preferably, this information should be extracted in an automated fashion without manual user interaction in real-time. Examples for the applications of real-time automated bioprocess monitoring include the monitoring and control of glucose and glutamine concentrations using online HPLC [35] and the simultaneous measurement of glucose, glycerol, ethanol, acetate, phosphate and ammonium using an online enzymatic robot [36]. Although very powerful, these methods require an automated bioreactor sampling port which constitutes a potential sterility hazard since these methods are invasive. Furthermore, constant withdrawal of on-line samples is a potential issue for small scale reactors in process development.

Real-time bioprocess monitoring devices based on spectroscopic methods, for example near infrared, mid- infrared and RAMAN, can be placed in-situ (inside the bioreactor). They offer the possibility of simultaneous and high frequency measurement of multiple components without constant withdrawal of on-line samples. However, the use of spectroscopic methods typically requires the use of chemometric tools for the establishment of robust calibration models. Bioreactor monitoring using spectroscopic methods and the processing of spectral data using chemometric methods is reviewed elsewhere [37].

Fourier transformation mid infrared spectroscopy (FT-IR) using an attenuated total reflection (ATR) interface is able to measure in the fingerprint region (1800-900 cm^-1^) of the mid-infrared range providing chemical information on many relevant molecules (e.g.: carbonic acids, sugars, alcohols etc.). It was successfully applied in bioprocessing for the measurement of carbon sources (arabinose, fructose, glucose, glycerol, methanol) and bioprocess metabolites (acetate and ethanol) [38-41]. ATR-FT-IR provides the advantages of fast (time range of 2 minutes) measurements of multiple components at a time. However, to extract quantitative information typically chemometric methods are required. For a thorough review see also [37].

### Soft sensors for bioprocess development

Software sensors or short “soft sensors” are mathematical tools that process readily available process signals to calculate unknown process variables. Roughly, they can be characterized in first-principle soft sensors that rely on first-principle relationships such as mass balancing and data driven approaches that demand experimentally obtained training data sets. The latter are mainly used for manufacturing where sufficient training data sets are available. In contrast, first-principle soft sensors demand little prior knowledge and no training data sets. Therefore, they increasingly find attention for process development purposes. First principle soft sensors on the basis of mass balances allow the real-time extraction of physiological information such as specific rates and yield coefficients without the need for off-line sampling, as demonstrated elsewhere [31,42,43]. This is especially important for bioprocess development in an industrial environment, where in contrast to universities frequent sampling intervals are typically not feasible.

### Aim of the contribution

This contribution aims at the presentation of a novel dynamic fed-batch method for the physiological investigation of microbial systems. The method allows the extraction of the relationship of the specific growth rate μ and temperature in induced conditions. This information is necessary for the science based basic design of recombinant bioprocesses. In order to comply with process developments’ demands for highly automated methods with little user interaction, the method was designed to allow real-time extraction of physiological information using a first-principle soft-sensor. Furthermore, a simple, but highly efficient PID control strategy for the control of residual substrate concentrations in fed-batch experiments based on simple process information is presented.

### Goals

Presentation of a novel method for the efficient and highly automated extraction of strain specific information through soft-sensors. Presentation of a novel and simple to be implemented control strategy for the control of residual substrate concentrations in fed-batch processes.

## Results

The determination of the temperature dependency of the specific growth rate (μ) was carried out in an efficient manner via a dynamic ramp experiment. The applicability of the method is demonstrated on a recombinant *E. coli* C41 bioprocess. In a second step, the method was automated by substituting at-line enzymatic glucose measurements by in-line ATR-FTIR measurements and the use of first-principle soft-sensors for the real-time extraction of physiological information. Prerequisite of the method is the control of the residual substrate concentration under non-limiting conditions. The design and in-silico testing of the developed control methodology to achieve this task is described in the following section.

### Design and in-silico performance of the PID control strategy

Control of the residual glucose concentration was achieved via a PID control strategy. Due to the non-linearity of bioprocesses PID controllers are typically not considered the first choice for bioprocess control applications. To overcome these limitations and avoid the re-adjustment of PID parameters, the control problem was approached as follows: Glucose consumption in fed-batch processes is proportional to base consumption, carbon dioxide evolution rate or other signals reflecting the total metabolic activity. The proportionality coefficient P of glucose consumption and the respective signal (actually a yield coefficient *Y*_*rs/ry,*_ whereas *r*_*S*_ is the glucose consumption rate and *r*_*y*_ is the signal rate) can change over time due to physiological changes of the host organisms, e.g. due to the consequences of metabolic load [44] and is typically unknown in early stages of process development. PID controllers aiming at the control of residual substrates typically act directly on the feed rate F for the control of the residual substrate concentration. Here, the PID control algorithm was designed to act on the proportionality coefficient *P*(*t*) instead of directly on the feed-rate. The feed rate *F*(*t*) is then calculated as product of the calculated proportionality coefficient and the proportionality signal *S* (Equation 2).

$$P\left(t\right)=K_{p}\;\cdot \;e\left(t\right)+K_{p}\;\int_{0}^{t}\;e\;\left(t\right)\;\mathrm{dt}+K_{d}\cdot \frac{d}{\mathrm{dt}}e\left(t\right)$$

Equation 1: PID algorithm for the proportionality coefficient *P*(*t*)

$$F\left(t\right)=P\left(t\right)\cdot \;S$$

Equation 2: Calculation of the feed rate as a function of signal S and the proportionality factor *P*(*t*).

An in-silico fed-batch model was used for the testing of the developed control strategy for the control of the residual substrate concentration. The model was run with a specific growth rate of 0.1 h^-1^ from biomass concentration 2 g/l to 50 g/l. 5% random noise was added to the “in-silico” glucose measurements (every 0.5 h), which served as the basis of the PID control strategy aiming at the control of the residual glucose concentration at 20 g/l. The base flow rate was chosen as proportionality signal. PID parameters were determined empirically and then held constant for all simulations.

**Figure 1:**
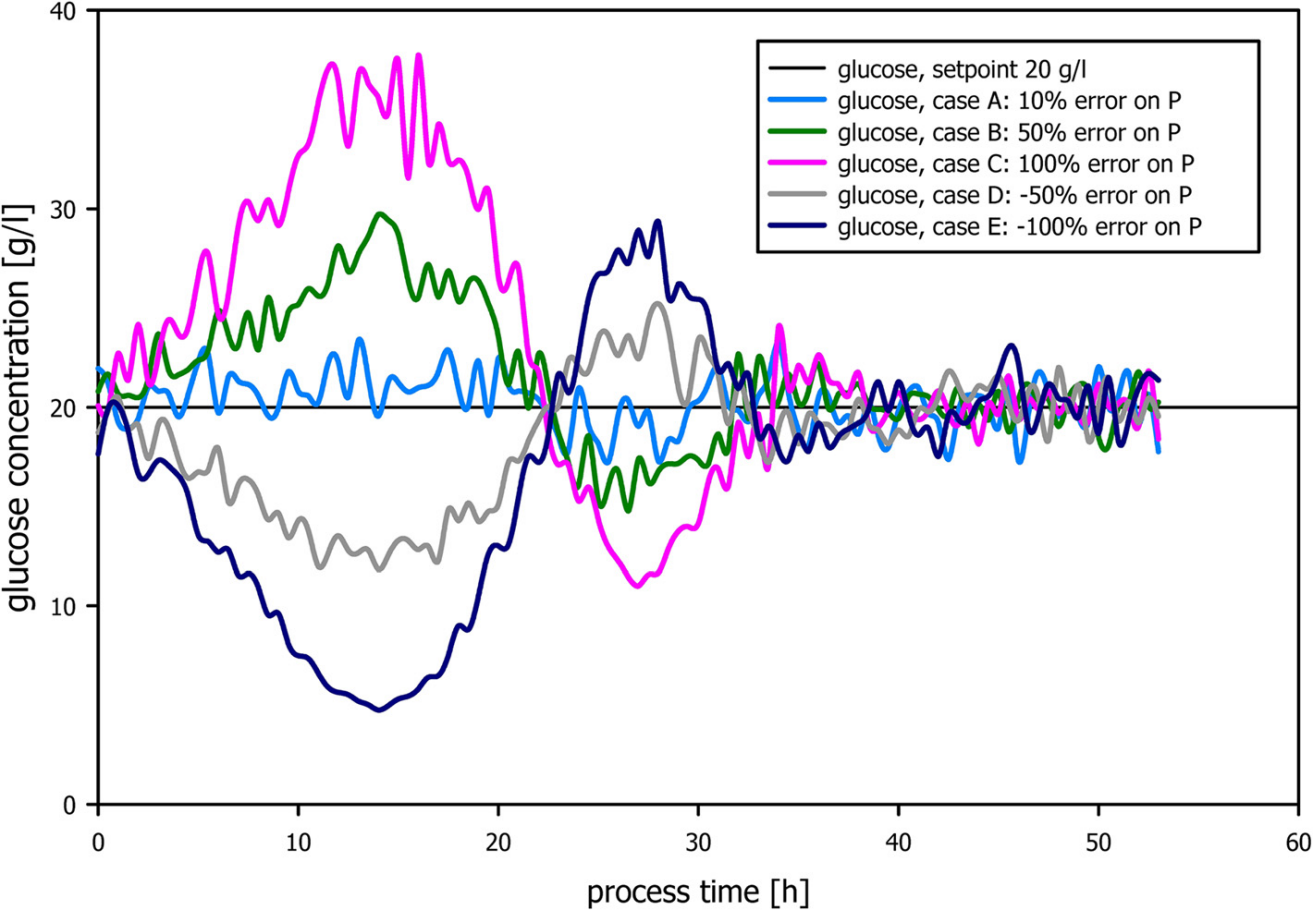
Build up response of the presented PID control strategy.

Since the proportionality coefficient *P* (here: the yield coefficient substrate consumption per base consumption because the base flow rate was chosen as proportionality signal) is not known prior to fermentation, the controller was tested for different initial values: 10%, 50% 100% -50% -100% (Figure 1). In general, the built-up response of the controller is equal to a typical PID controller. In case the initial proportionality constant is off by 10% (Figure 1, Case A), the controller efficiently controls the residual substrate concentration efficiently at 20 ± 3 g/l. Higher deviations from the true value (Cases B to E) show higher deviations from the set-points and a slower build up response.

On the basis of the in-silico results (initial transient behaviour for the case of an unknown yield coefficient, long-term stability) it was concluded that the presented formulation of a PID control strategy is applicable for the control of residual substrate concentrations in case a reference measurement (e.g. at-line enzymatic analysis or in-line spectrometric measurement) is available and a good initial proportionality factor (less than 50% deviation from the true value) can be delivered to the PID controller. Tuning of PID parameters can be efficiently done via in-silico simulation.

### Performance of the presented method: *E. coli* C41 pBMPpeT- non-induced conditions

**Figure 2:**
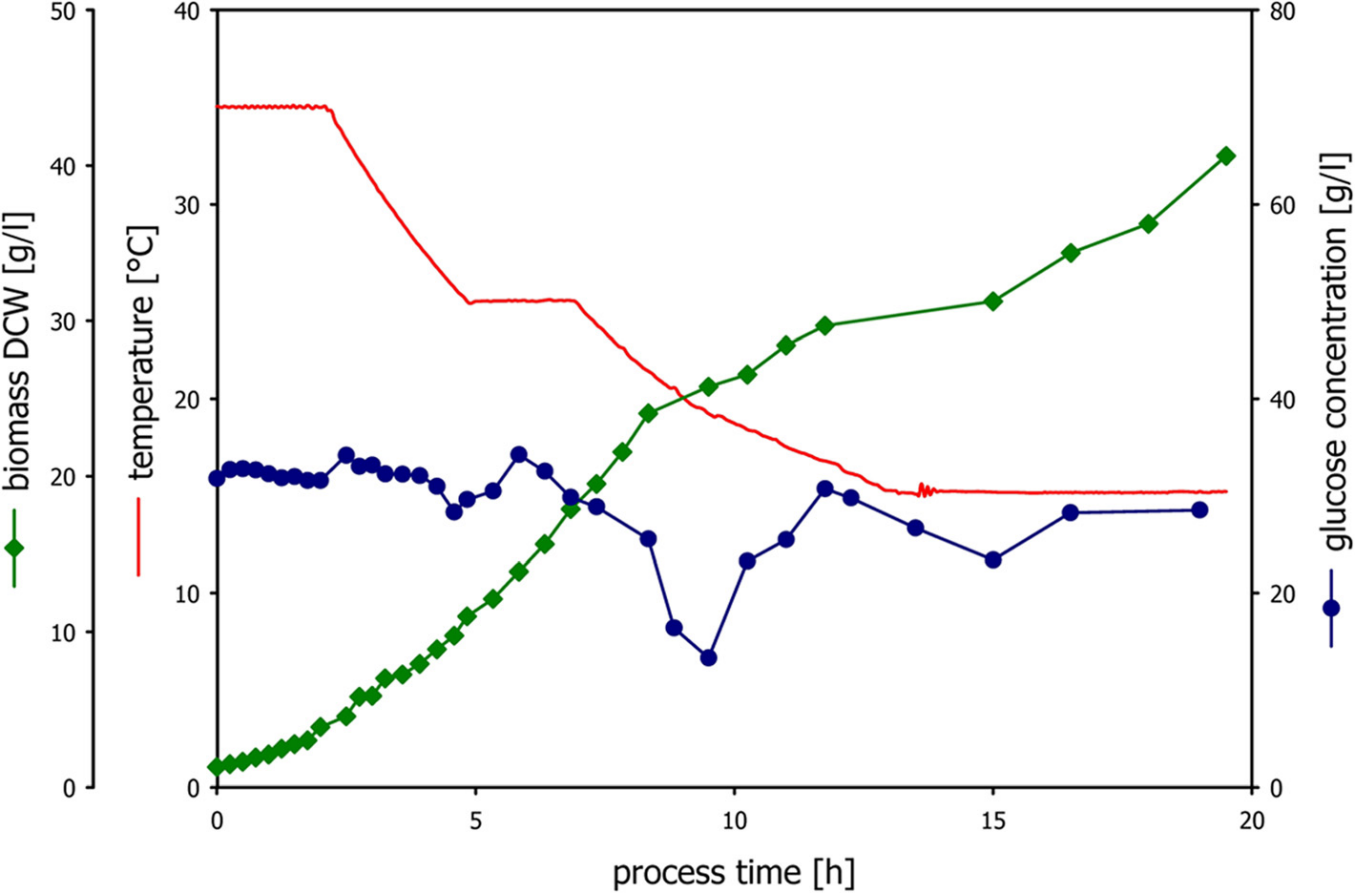
**Dynamic investigation of the specific growth rate as a function of temperature.** Residual glucose was controlled at 20 g/l using at-line enzymatic measurements. The culture was submitted to dynamic ramps in temperature. Biomass growth was monitored through off-line sampling.

The bioreactor containing 4 liters batch medium (20 g/l glucose) was inoculated with 100 ml preculture and grown for 8 hours at 35°C to obtain a quantifiable amount of biomass and biologic activity. Subsequently, the PID control strategy with the base feed rate as proportionality factor was started to control the residual substrate concentration at 20 g/l, as described in the previous section. Initial proportionality coefficient was estimated from batch data (proportionality coefficient of base and feed flow rate). Sampling intervals were chosen according to Equation 4 and adapted as a function of the estimated specific growth rate. Residual glucose concentrations were measured enzymatically at-line and provided immediately to the PID controller. The temperature changed dynamically in a range from 35 to 15°C within a total time of 20 hours (see Figure 2). Biomass dry cell weight estimation from 2 to 10 g/l was done via OD correlation. Biomass dry cell weight was determined gravimetrically in a range from 10 to 70 g/l. Between temperature set-points the temperature was changed transiently, allowing for the extraction of the information μ f(T) at the temperature set-points as well as for the temperature transients. Residual glucose was successfully controlled at non-limiting conditions during the whole process.

**Figure 3:**
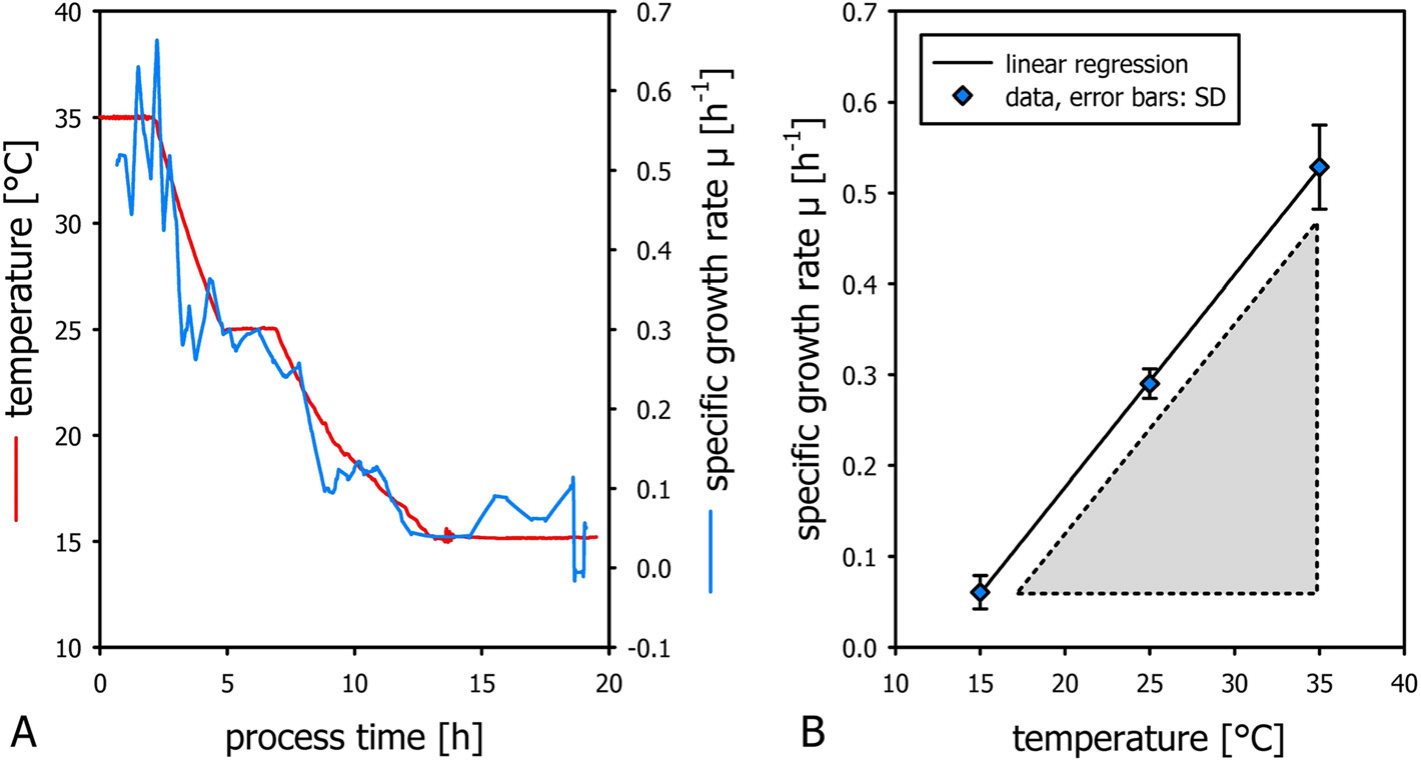
**Specific growth rate as a function of temperature in non-induced conditions.** Off-line biomass dry cell weight concentrations were used for the calculation of the specific growth rate μ **(A).** μ was found to correlate with cultivation temperature (T) throughout the whole fermentation process. The function μ f(T) was obtained via linear regression **(B)**.

Specific growth rates were calculated on the basis of biomass dry cell weight measurements and found to decrease linearly as a function of temperature as illustrated in Figure 3A. Specific growth rates calculated for temperature set-points were averaged and linearly regressed to find the strain specific function μ f(T) for the strain under investigation (Figure 3B).

### FTIR assisted control and soft sensor assisted real-time extraction of information in induced conditions

The method described in in the previous section is heavily dependent upon frequent off-line sampling and therefore difficult to be applicable in a pharmaceutical bioprocess development environment where a high degree of automation and little manual interaction is necessary for cost efficiency. Hence, the method was automated in respect to control via the use of in-line ATR-FTIR spectroscopy to substitute the at-line measurement of residual glucose, as well as in respect to information extraction via the use of a first principle soft sensor for the estimation of the specific growth rate (desired information) as well the biomass concentration.

**Figure 4:**
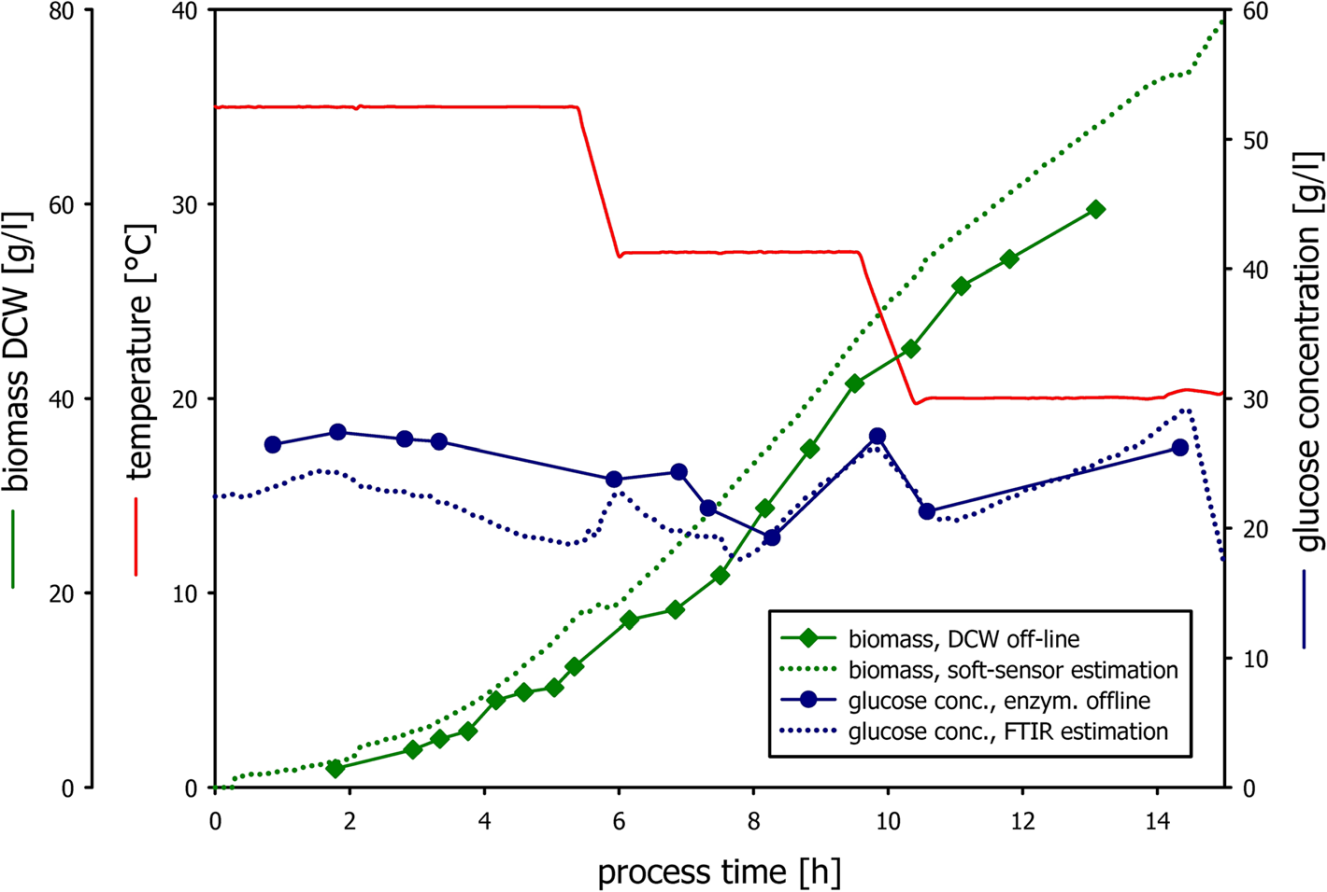
**Dynamic investigation of the specific growth rate as a function of temperature for induced process conditions and automated extraction of information.** Residual glucose was controlled at 20 g/l via an in-line FTIR control strategy and cross checked by off-line enzymatic measurements. The culture was submitted to dynamic ramps in temperature. Biomass growth was monitored through off-line sampling and estimated via the soft sensor.

Results are depicted in Figure 4. The culture was grown to 5 g/l and subsequently induced by 10 mM IPTG. Glucose concentrations were controlled at non-limiting conditions. Residual glucose concentrations predicted via in-line FTIR measurements showed a maximum deviation of ±5 g/l. A first-principle rate based soft sensor was used for the estimation of the biomass formation rate as well as specific rates and yield coefficients. The temperature was changed dynamically in a range of 35°C to 20°C. Estimated biomass concentrations and measured biomass concentrations (biomass dry cell weight) are depicted in Figure 4.

**Figure 5:**
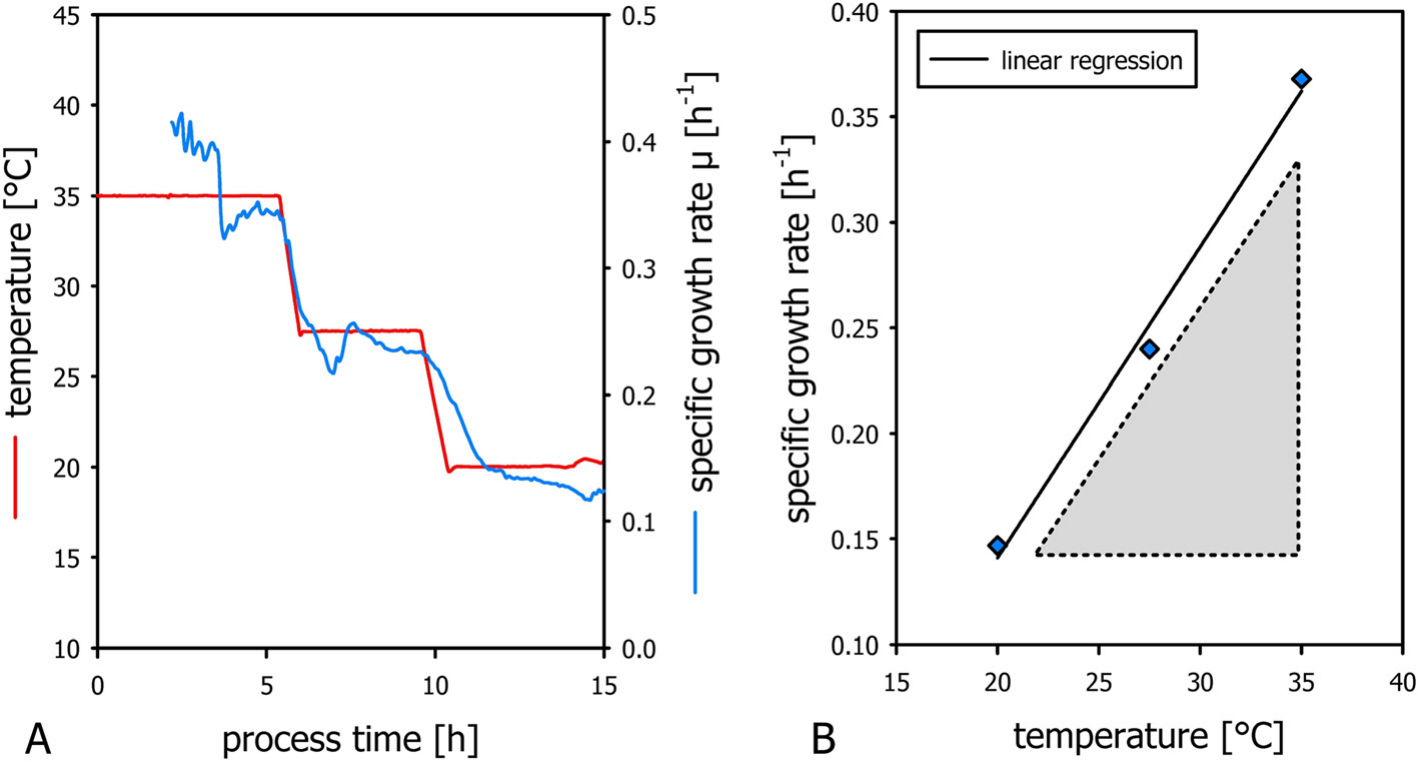
**Specific growth rate as a function of temperature in induced conditions.** The soft sensor was used for the estimation of the specific growth rate μ **(A).** μ was found to correlate with cultivation temperature (T) throughout the whole fermentation process. The relationship of the specific growth rate as a function of temperature can be read from the plot μ versus temperature **(B)**.

Specific growth rates automatically estimated from the soft-sensor without the need for off-line measurements are depicted in Figure 5A. From this information, the strain specific function μ f(T) can be extracted (Figure 5B).

## Discussion

### Induced state specific growth rates

The investigation of the dependency of the specific growth rate μ on parameters such as the cultivation temperature has been a primary interest of microbial research [45-48]. The reduction of the specific growth rate μ as a results of recombinant protein expression due to metabolic load is discussed in several contributions [44,49]. Recombinant systems are prone to energy/precursor drain due to recombinant protein expression, a phenomenon often referred to as “metabolic load” or “metabolic burden” [49,50].

As expected, in the presented study the temperature dependency of the specific growth rate was found to differ strongly under induced and non-induced conditions (see Figure 3 and Figure 5). This can be attributed to the effect of metabolic load due to recombinant protein production. This underpins that repetitive batch experimentation, chemostat and shake flask experimention running under non-induced conditions are not applicable for the physiological investigation of induced recombinant bioprocesses. In the presented method, cultivation temperature was varied from high to low set-points. Consequently, specific growth rates were also varied from high to low values. Hence, high specific growth rates were run at low biomass concentrations in the beginning of the experiments and low specific growth rates were run in the end of the experiment where biomass concentrations are high. This has advantages in respect to avoiding to exceed the maximum oxygen transfer rate, the maximum heat transfer rate and the accumulation of overflow metabolites, as also discussed elsewhere [22].

### Maximum induced state metabolic capabilities as prerequisite for bioprocess design and DoE based bioprocess optimization

The presented method allows to determine the strain specific function μ_max_ f(T) in the induced state in an automated and efficient way. The area under the function μ f(T) highlighted in Figure 3, B and Figure 5, B spans the space of process parameters that are feasible from an physiological point of view. The respective function can be considered a bioprocess design boundary: Exceeding the maximum specific growth rate would lead to uncontrolled accumulation of substrate. This is especially important in multivariate studies aiming at the investigation of the impact of temperature and specific growth rates on process performance.

### Measurement and control of residual substrate concentrations in fed-batch processes

In this study, a combination of a first-principle soft-sensor with spectroscopic estimation of glucose was used to control glucose at a non-limiting level. A very simple linear calibration model based on a single band in the mid IR spectrum was used for the estimation of the glucose concentration, since within the early stages of process development there is typically insufficient data for multivariate calibration models [41,51]. Such models are often more capable of quantifying multiple components at once, with a lower error of prediction, compared to the simple model used within this contribution. For the task in this contribution (control residual substrate concentration at non-limiting conditions), the linear calibration models turned out to be sufficient. However, a considerable improvement of residual glucose estimation can be expected by the use of multivariate calibration models. Furthermore, the use of spectra libraries [52] can be a promising alternative.

Within this contribution the control of the residual substrate concentration was achieved using a simple PID control strategy in combination with ATR-FTIR measurements of residual glucose. In contrast to traditional approaches where the PID controller acts directly on the feed-rate, it was designed to act on a proportionality factor (P) as described in Equation 2. P can be interpreted as the yield coefficient of carbon source conversion in respect to the ammonia conversion. This is to our knowledge a novel approach which was proven that it is applicable to be applicable both in in-silico simulations (Figure 1) as well as in real *E. coli* bioprocesses (Figure 2 and Figure 4).

In general, the control of the glucose concentration in bacterial fed-batch processes is a highly challenging task due to high glucose conversion rates and high process dynamics. This control approach is atypical for bacterial fed-batch fermentations, since they are generally run under substrate limitation. However, control of the residual substrate concentration is desired in recombinant mammalian cell cultures since the glucose concentration affects the glycosylation pattern of the recombinant products [35,53]. Proportional integral (PI) or proportional integral derivative (PID) controllers are simple and easily implementable algorithms for linear systems [54]. However, bioprocesses are highly non-linear by nature and show high process dynamics, typically discarding PI or PID approaches. Therefore, control challenges such as the control of residual glucose concentrations in fed-batch experiments are typically approached by more sophisticated controllers such as adaptive controllers, Kalman Filters or Neural Network or Fuzzy control approaches [35,55]. However, the necessity for process models or training data sets discards these control approaches for process development purposes, where the necessary information basis (e.g.: a suitable process model) is typically not available. The presented approach using a PID controller to act indirectly on the feed rate can be considered a promising alternative for applications where no or little prior knowledge on the system is available.

### Process development using a toolset of dynamic methods, soft-sensors and parallel bioprocessing

We anticipate that soft-sensors, soft-sensor assisted bioprocess control strategies and the use of advanced on-line analytics will play key roles for the acceleration of bioprocess development as required by the biotech industry. We believe these methods are especially powerful in combination with parallel processing [56,57]. Within this contribution, we demonstrate the benefits of the combined use of advanced process analytics (ATR-FTIR), first-principle soft sensing and dynamic experimentation, aiming at the fast and automated extraction of physiological information. Furthermore, due to limited user interaction and a high degree of automation, the method can be used for multiple bioreactors simultaneously, as required for parallel bioprocessing applications.

The presented method complements the toolset of dynamic experiments available and sets directions for future progress in the field efficient bioprocess development.

## Conclusions

Dynamic methods provide fast and efficient alternatives to classical chemostat-, shake flask and repetitive batch driven physiological investigation of microbial systems.

In order to apply dynamic methods in industrial process development, process automation in respect to information extraction and process control is necessary.

First-principle soft sensors are efficient tools for the extraction of strain specific parameters from dynamic bioprocesses and can be used to reduce manual sampling effort.

The presented method allows the investigation of the relationship of the specific growth rate as a function of the temperature in an efficient and automated fashion without the need for sampling both for induced and non-induced bioprocesses.

## Methods

### Strain

*E. coli* C41 (F^–^ ompT hsdSB (rB^-^ mB^-^) gal dcm (DE3); Lucigene, Middleton, WI, USA) with the plasmid phBMPpET encoding for human bone morphogenetic protein 2 (rhBMP-2) was used. Strains and plasmids were gratefully provided by BIRD-C GmbH & Co KG, Kritzendorf, Austria and Morphoplant GmbH, Bochum, Germany.

### Media

A defined minimal medium with D-glucose as main carbon source (batch medium glucose concentration: 20 g/l; fed-batch medium glucose concentration 400 g/l) as described in detail elsewhere [58] was used.

### Bioreactor setup

A Techfors-S bioreactor (Infors, Bottmingen, Switzerland) with 10 l working volume was used. For gravimetric flow quantification, feed and base bottles were placed on scales (Sartorius, Göttingen, Germany). A Techfors-S integrated analogue pump was used for the addition of glucose. The bioreactor was equipped with probes for the measurement of dissolved oxygen (Hamilton, Reno, USA), pH (Hamilton, Reno, USA) and head pressure (Keller, Winterthur, Switzerland). CO_2_ and O_2_ in the off-gas were measured by a gas analyzer (Müller Systems AG, Egg, Switzerland), based on non-dispersive infra-red (CO_2_) and paramagnetic (O_2_) principle. All signals were collected by the process information management system (PIMS) Lucullus (Secure Cell, Schlieren, Switzerland).

### Fermentation parameters

Dissolved oxygen levels (DO_2_) were kept above 40% saturation (100% saturation were set before inoculation at 35°C, 0.3 bar gauge, pH 7.2). The pH was kept constant at 7.2 by adding 12.5% NH_4_OH, which also served as nitrogen source. Dynamic temperature ramps and feeding profiles are described in the results section.

### Biomass dry weight concentration

2 ml of the cell suspension were centrifuged (RZB 5171, 10 min, 4°C) in pre-weighted glass tubes, washed twice using distilled water and dried at 105°C for 72 hours. The biomass dry weight concentration (BDW) was determined in duplicate.

### Metabolite concentrations

Cell-free supernatant samples for the determination of residual substrate concentrations were taken from the vessel using an in-line ceramic 0.2 μm filtration probe (IBA, Heiligenstadt, Germany). Off-line and at-line concentrations in the supernatant were measured enzymatically using a photometric robot (CuBiAn XC; Opto-Cell, Germany).

### Soft sensor rate calculation, reconciliation, biomass estimation, in-silico simulations

**Figure 6:**
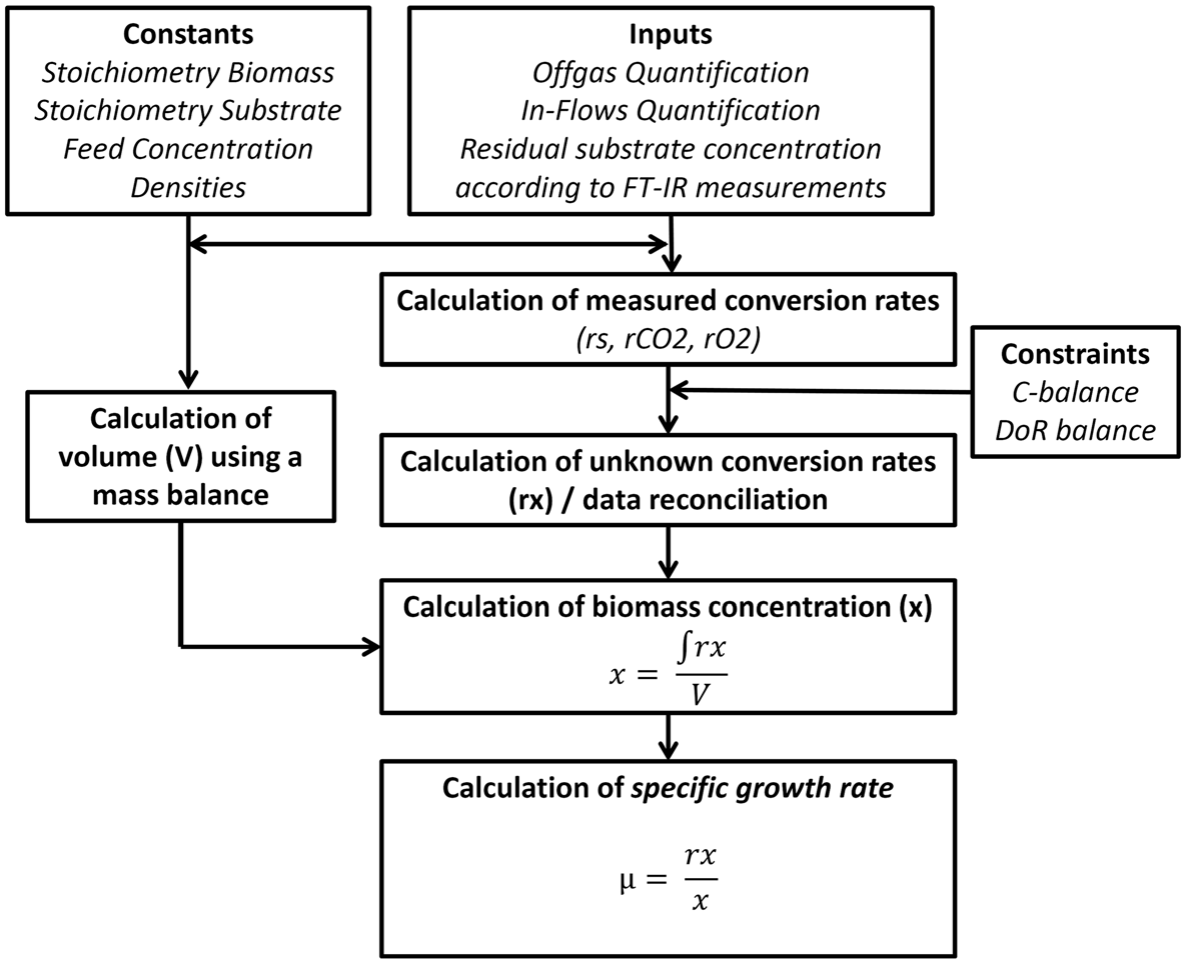
Illustration of the soft-sensing strategy to extract automatically information in the form of specific rates (specific growth rates).

A first principle soft sensor designed for process development as described in detail elsewhere [31,43] was used within this contribution. The soft-sensing concept is illustrated in Figure 6. In short, the soft-sensor uses inputs from the process such as offgas O_2_ and CO_2_ concentrations using off-gas analysis, in-flows quantification using mass flow controllers, residual substrate measurements using FT-IR and a set of constants to calculate conversion rates of substrate (rs), carbon dioxide (rCO2), and oxygen (rO2). Changes in the residual substrate concentration as measured via on-line FTIR were made available to the soft-sensor. The unknown conversion rate of the biomass formation (rx) was estimated using the carbon and degree of reduction (DoR) balance as linear constraints as described elsewhere [59]. The biomass concentration is obtained via integration of the estimated biomass formation rate with time. Subsequently, the specific growth rate (see Equation 3), specific substrate uptake rates of carbon sources as well as yield coefficients can be calculated as described in detail elsewhere [31].

$$\mu \;=\frac{\mathrm{rx}}{x}$$

Equation 3: Calculation of the specific growth (μ) rate as a function of the estimated biomass formation rate (rx) and the estimated biomass concentration (x) using the soft sensor.

Numerical simulations of the PID control strategy were carried out with MATLAB 2012a (Mathworks, Natick, Massachusetts, USA) and Excel (Microsoft, Redmond, Washington). The in-silico model was adapted from Wechelberger et al. (2013) [60].

### In-line residual substrate measurement

An ATR-FTIR spectrometer (ReactIR, Columbus, Ohio, USA) interfaced with the bioreactor via a 25 mm Ingold nozzle was used. Infrared spectra (256 scans) were measured in intervals of 2 min and used for the estimation of residual glucose concentrations *via* a simple linear calibration model using a main absorption band of glucose (1036 cm^-1^) in the mid infrared range. To account for baseline drifts, the spectra where off-set corrected at 1807 cm^-1^, since there were no components with absorption at this wave number in the culture broth. Calibration spectra are displayed in Additional file 1.

### Real-time data processing

IC Quant (Mettler Toledo, Columbus, Ohio, USA) was used for the real time computation of spectra for the estimation of the residual glucose concentration. Glucose concentrations were imported to Lucullus PIMS as text files (Secure Cell, Schlieren, Switzerland) and delivered to the soft-sensor. The soft-sensor was implemented using the Sim-Fit tool of the Lucullus PIMS (Secure Cell, Schlieren, Switzerland).

### Definition of sampling intervals

The signal to noise ratio of timely resolved rate-based information (e.g. the specific growth rate μ) calculated from off-line data is dependent upon the error on the respective offline measurement, the variation (signal) as well as the time window [60]. To achieve comparable signal to noise ratios from off-line data in all process phases, sampling intervals were calculated according to Equation 4 [60] with a desired signal to noise ratio (SNR) of 12 and an expected error (BM) on the biomass measurements of 1%.

$$\text{Sampling}\;\text{Interval}=\frac{\text{SNR}*\text{error}\;\left(\text{BM}\right)}{\mu *67}$$

Equation 4: Estimation of the sampling interval based on signal to noise ratio (SNR), the specific growth rate μ and the expected error (BM) on the biomass measurements.

## Abbreviations

μ: Specific growth rate [h^-1^]; T: Temperature [°C]; FTIR: Fourier transformation infrared; CPP: Critical process parameter; CQA: Critical quality attribute; PAT: Process analytical technology; qs: Specific substrate uptake rate [g/(gh)]; SNR: Signal to noise ratio; error (BM): Error in biomass quantification [%]; DOE: Design of experiments; Y(x/s).: Biomass yield coefficient [g/g]; rs: Substrate converstion rate [g/(lh)]; rx: Biomass formation rate [g/(lh)]; P: Proportionality factor; E: Control deviation; t: Time; PID: Proportional integral derivative; K: Coefficient of PID controller; F: Flow rate [g/h]; S: Signal as basis of control strategy; BDW: Biomass dry cell weight [g/l].

## Competing interest

The authors declare that they have no competing interests.

## Authors’ contributions

PS and CH jointly conceived the study. PS designed the experiments, drafted the manuscript and conducted data analysis and data interpretation. TL participated in the design and coordination of the study and helped to draft the manuscript. PW participated in the design and application of the soft sensing strategy. AM conceived and performed the analytical concept. All authors read and approved the final manuscript.

## Supplementary Material

Additional file 1 Fourier Transformation Infrared Spectra. Click here for file
