# Supplementary material for: A dynamic method for the investigation of induced state metabolic capacities as a function of temperature
Source: Microb Cell Fact. 2013 Oct 15;12:94. doi: 10.1186/1475-2859-12-94 (PMC4015482; doi:10.1186/1475-2859-12-94)
Supplement: Additional file 1 — Fourier Transformation Infrared Spectra. [file 1475-2859-12-94-S1.pdf]

Additional File 1: Fourier Transformation Infrared Spectra

Supplementary Figure 1 shows the spectrum of the culture broth before and after the addition of 20 g/l glucose. Cultivation broth background was subtracted at the start of the experiment. The most prominent band at 1036cm-1 was chosen for a simple two-point calibration model for estimation of glucose within this contribution. To account for baseline drifts, the spectra were off-set corrected at 1807 cm-1, since there were no components with absorption at this wave number in the culture broth.

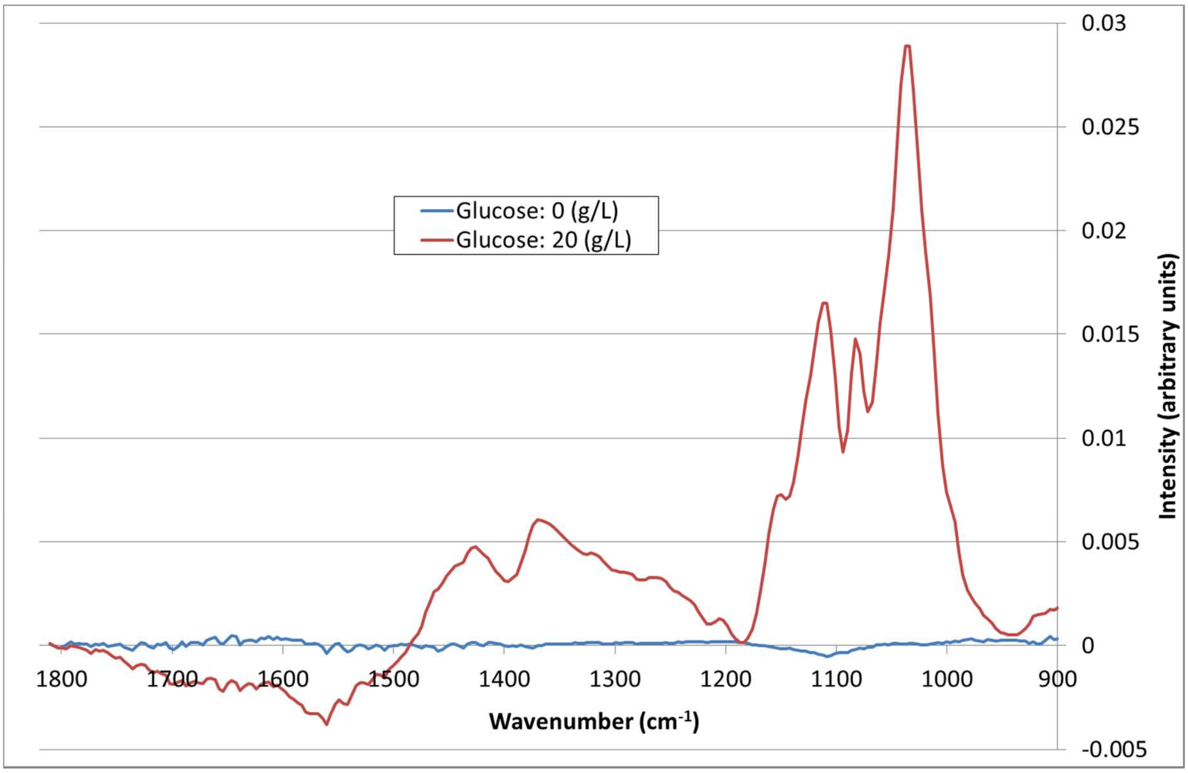

Supplementary Figure 1: Spectrum of the culture broth before (blue line) and after (red line) the addition of 20 g/l glucose
